# Spatiotemporal changes in river flow seasonality and predictability using Colwell indices in Northwestern Iran

**DOI:** 10.1038/s41598-025-33205-1

**Published:** 2025-12-23

**Authors:** Parvin Momenian, Nazila Alaei, Raoof Mostafazadeh

**Affiliations:** 1 School of Plant, Environmental and Soil Sciences, Louisiana State University, Baton Rouge, 9873002863 USA; 2 Department of Range and Watershed Management, Faculty of Natural Resources, Urmia University, Urmia, 5756151818 Iran; 3 Department of Natural Resources, Faculty of Agriculture and Natural Resources, Member of Water Management Research Center, University of Mohaghegh Ardabili, Ardabil, 5951816687 Iran

**Keywords:** Seasonal flow variability, Streamflow dynamics, Colwell indices, Periodicity, Climate sciences, Environmental sciences, Hydrology

## Abstract

The study aimed to examine seasonal-spatial changes in river flow regimes in northwestern Iran using Colwell indices, which assess the regularity and seasonal pattern of flow through three components: Predictability, Constancy, and Contingency. This study is the first to apply Colwell indices across rivers in northwestern Iran, revealing varied trends influenced by climate and human activities. In this study, river flow data from various rivers in northwestern Iran with climate diversity were used to analyze the time and spatial changes of river flow regimes. In this regard, using the Colwell indicators, the variations of the different components of the river flow regime were evaluated from three main aspects including Predictability (P), Constancy (C) and Contingency (M) on different daily, monthly and seasonal time scales. The results showed that the ArbabKandi river had the highest Predictability and Constancy in its currents with values of P_MaxM = 0.739 and C_MaxM = 0.651 and Nir with values of P_MinS = 0.64 and C_MinS = 0.42, while the dostbeglo and Hir Rivers had the lowest values. Analysis of maximum, minimum and average of Colwell indicators of the flow regime showed significant differences between rivers due to the influence of various climatic factors or human exploitation. Trend analysis in Ardabil Province based on the Modified Mann-Kendall test reveals significant river flow decreases at stations such as Samian (S = − 649), Borran (S = − 639), Dostbeglo (S = − 643), and Mashiran (S = − 665), while Abgarm (*p* = 0.55), AhleIman (*p* = 0.50), Hir (*p* = 0.22), and ShamsAbad (*p* = 0.15) show no significant trends. Moderate but significant declines are observed at Arbabkandi, Barough, Gilandeh, Hajahmadkandi, Nir, Nuran and Viladaragh (*p* < 0.05). The study emphasizes the importance of understanding the variability of river flow regimes and river flow discharge values and can help improve water resource management strategies and better predict future changes.

## Introduction

### Background

Water resource management is a complex subject that has consistently garnered attention due to its susceptibility to various factors. Studies have shown that changes in climatic variables affect hydrological cycles, agricultural yields, plant-animal ecosystems, and socio-economic conditions^1,2^. Evaluating temporal changes in river flow and water yield patterns significantly influences effective water resource management. Implementing appropriate management decisions regarding water resource utilization requires considering the impact of climate fluctuations and human factors on river flow regimes and runoff production from watersheds^3,4^. One of the indicators used to diagnose and determine the controlling and influential processes on runoff production and temporal changes is the seasonality of runoff regimes^5^. Hydrological indices, such as temporal changes in river flow, vary significantly across different regions and climates. These changes in river flow and hydrological regimes are influenced by climatic components, surface, and subsurface processes^6^. While most studies on flow regimes have focused on individual runoff events^7^, our understanding of seasonal changes in runoff generation and river flow pathways remains limited^8^. Seasonal changes in precipitation and temperature can lead to significant alterations in the timing of peak flows within a watershed^9^. Mountainous watersheds are often affected by the timing and amount of snowmelt, significantly influencing runoff seasonality^10^. In essence, hydrological processes in mountainous watersheds exhibit significant spatiotemporal variability^8^. Changes in climate can significantly affect snowmelt characteristics, potentially resulting in seasonal variations in runoff^11,12^. The role of snow cover and snowmelt in runoff changes, summer low flows, and water supply is often influenced by the timing of snowmelt, snow water equivalent, and the percentage of precipitation falling as snow^13,14^. Low flows are typically seasonal, and droughts affected by their changes often occur during low-flow seasons, to some extent predictable^15,16^. From a practical standpoint, seasonal flow regimes are crucial for water resource management, drinking water supply, agriculture, hydropower generation, and river ecosystem viability^17–20^. Changes in the pattern of runoff variations (including frequency, magnitude, rate of change, and timing of flood and low-flow events) and the trend of seasonal runoff changes can be evaluated based on recorded data^21–23^. Due to the complexity of watershed systems and drainage networks, calculating and quantifying various indices related to river flow regimes can assist managers and policymakers in making optimal decisions regarding water allocation and management^24^. Numerous studies worldwide have examined temporal changes and the seasonality of river flow. For instance, Devineni and Arumugam^9^ investigated the relationship between temperature, precipitation, and monthly runoff in the eastern United States. They found significant differences in seasonal flow despite uniform monthly precipitation throughout the year. According to their analysis of the seasonality index, in areas with uniform precipitation distribution throughout the seasons, river flow occurs during the snowmelt season, whereas in regions with uniform rainfall distribution, the targeted index occurs based on seasonal storage.

### Literature review

La Torre Torres et al.^25^ examined seasonal relationships between rainfall patterns and runoff using precipitation and base flow data from 1964 to 1976 in a forested watershed. Their results showed that runoff is primarily controlled by rainfall amounts and antecedent soil moisture conditions, leading to significant seasonal variations in runoff production due to differences in evapotranspiration, soil moisture, and precipitation distribution. Peterson et al.^26^ investigated the seasonality of streamflow and its controlling processes in the United States using climate and land surface characteristics. Their study revealed that monthly cycles of moisture and energy in the eastern United States are influential factors in the spatial variability between average monthly peak precipitation and annual precipitation and that the spatial variability of runoff seasonality is strongly related to basin aridity and precipitation seasonality. Schneeberger et al.^12^ examined the impact of climate change on seasonal runoff in a mountainous watershed in Australia. Using various climate variables for modeling future climate runoff, their results indicated that changes in temperature and precipitation patterns can significantly affect seasonal runoff in the target reservoir basin. Wang et al.^27^ investigated the susceptibility of two indices, Maximum Daily Difference (MDD) and accumulated direct discharge (ADD), to extreme weather events and runoff in a cold climate region in China. They found that based on average daily discharge from 1961 to 2007, increasing glacier coverage in the area leads to a more significant increase in runoff. Their analysis used a regression approach to assess the relationships between runoff indices in summer and variables related to precipitation and temperature. Guadry et al.^5^ analyzed daily precipitation and discharge data in Peru and Austria, demonstrating higher runoff seasonality variability in Peru due to climate and topographic diversity, while in Austria, snowmelt effects lead to greater runoff seasonality than precipitation. Liu et al.^28^ presented an analysis of the variance of runoff based on factors such as temporal variance of runoff (RV), precipitation (P), potential evapotranspiration (E0), total water storage changes, and other factors in the Budyko equation. By evaluating the seasonality of climatic components, land cover, and human effects at intra-annual, inter-annual, and decadal scales, it was determined that precipitation controls RV values in many parts of the world at different temporal scales. Moreover, P showed a greater effect on RV in humid basins and at multi-year scales, while other factors exhibited a greater impact in dry basins. Hansford et al.^29^ introduced numerical criteria to assess the relationship between river flow patterns and climate, categorizing river flow patterns into four different statistical groups (a) stable hydrological rivers, (b) single-storm-controlled variable discharge rivers, (c) rivers with seasonal hydrology, and (d) highly irregular hydrological rivers based on flood magnitude, hydrograph shape, and inter-annual discharge variability. Gnann et al.^30^ demonstrated the effect of seasonal precipitation climate input variables on watershed runoff analysis in Britain and the United States. They showed that interpretable hydrological patterns are a function of climatic characteristics and watershed features, focusing on analyzing sinusoidal wave changes in dry and wet basins. Their results suggest that seasonality indices have the potential to be used for watershed classification and modeling in data-sparse basins. Jenicek and Ledyinka^10^ investigated the impact of changing precipitation from snow to rain on seasonal and annual runoff. They examined how the effects of snow storage in spring and early summer influence runoff, concluding that 17%-24% of total runoff is attributed to snowmelt, while snow contributes to 12%-37% of total precipitation, with less snowfall resulting in lower runoff. Additionally, rapid snowmelt reduces groundwater recharge, and high evapotranspiration leads to low summer flows. Hanus et al.^31^ found basin elevation linked to changes in runoff timing and annual minimum flow but weakly related to annual maximum flow in six Austrian watersheds. According to the literature, there exists a research gap concerning the comprehensive mapping and analysis of spatial and temporal changes in runoff seasonality and flow regime characteristics. While existing studies have provided valuable indications into the factors influencing runoff patterns, there is limited research focusing on assessing river flow regime using Colwell indices in regions with diverse affecting factors such as climatic factors, land use, and human activities. Reviewed studies analyzed various river flow aspects including seasonal variability^25,26^, climate change impacts on runoff^12,31^, effects of extreme events and glacier coverage^27^, snowmelt influence^5,10^, and runoff variability related to climate and land cover^28–30^. These studies showed seasonality shifts and climate-driven flow changes, supporting our Colwell-based runoff analysis. Unlike IHA or RVA, which emphasize flow magnitude, timing and frequency, Colwell indices directly measure the regularity, stability and seasonal recurrence of river flows in a simple, unitless form. This makes them useful for policymakers needing a single comparable metric to detect loss of seasonality caused by climate change or dam operation. Understanding low contingency (loss of seasonal rhythm) or low constancy (unstable flows) provides more practical guidance for reservoir operation, irrigation planning and drought management than only analyzing extreme flows. Colwell indices also standardize hydrological information (0–1), enabling comparison between basins and management conditions. Although many studies address seasonality indices and river flow changes, detailed analysis of spatial and temporal variability in regions affected by diverse climate, land use, and human activity is limited. Common indices often miss complex interactions between factors. This study fills these gaps by using Colwell indices, which better quantify predictability, constancy, and seasonality, providing a deeper understanding through comprehensive analysis of hydrological data from Ardabil province.

### Scope and objective

The literature lacks in-depth investigations into the mapping and temporal trend of seasonality indices. By incorporating spatial and temporal variability in runoff seasonality and flow regime characteristics, policymakers and stakeholders can make informed decisions to mitigate the impacts of climate change and ensure the sustainable use of water resources in various regions. Among the indices that could assist in better water resource and watershed management are the Colwell indices. Unlike other available indices, Colwell indices provide unit values of parameters for detecting river fluctuations. Although Colwell indices are flexible in application, the underlying hydrological data typically require standard preprocessing, such as handling missing values and ensuring data quality, to ensure reliable results. The primary reasons for selecting Colwell indices include analyzing predictability of events and changes in river seasonal flow and examining the temporal persistence of river flow pulses^24^. Colwell indices quantitatively describe river flow seasonality. Predictability (P) shows how regular and repeatable flow patterns are by combining constancy and contingency. Constancy (C) measures how stable the flow is throughout the year. Contingency (M) reflects how seasonal flow patterns repeat across years. Together, these indices capture both the stability and seasonal changes in river flow. Ardabil Province was selected for its diverse geography and climate, causing significant variability in river flow patterns^32^. Located in northwestern Iran, with both mountainous and plain areas, it is ideal for studying spatiotemporal river flow seasonality. These rivers are vital for agriculture and water supply, making flow understanding crucial for management^33^. Ardabil’s vulnerability to climate change and its position between the Caspian Sea and Iranian plateau offer varied conditions for analyzing seasonal flow using Colwell indices. In recent years, climate change, diverse water uses, and human activities like dam construction have significantly altered river flow regimes in Ardabil Province. However, studies on flow predictability, constancy, and seasonality remain limited, especially in areas with varied climates and topography. This study addresses this gap by applying Colwell indices to better understand river flow dynamics and support adaptive water resource management.

Northwest Iran, positioned between the humid Caspian and central semi-arid climates, features a complex climatic gradient that has been largely overlooked in previous studies. Unlike earlier research focused on a single time scale or flow metric, this study examines multiple temporal scales (monthly/seasonal) and various flow conditions (mean, median, minimum, maximum) using Colwell’s indices. This study performs a comprehensive spatiotemporal analysis of river flow seasonality and predictability across 28 rivers in northwestern Iran’s Ardabil Province, using long-term daily discharge data. We applied Colwell’s indices across multiple temporal scales (monthly/seasonal) and flow metrics (mean, median, minimum, maximum). The research presents three key innovations: (1) first regional-scale application of Colwell’s indices, providing integrated analysis of flow regime dynamics across complex topographic and climatic gradients; (2) establishment of a crucial baseline for understanding flow stability and predictability in a data-limited region; and (3) integration of trend analysis to detect temporal changes and identify vulnerable river systems. The aim of this study is to determine various characteristics of changing river flow regimes based on calculating seasonality using Colwell indices. Evaluating spatial and temporal changes in river flow regime characteristics and the influencing factors in different regions with a variety of climatic, topographic, and vegetative variables in northwest Iran is another objective of the current research.

## Materials and methods

### Geographical setting of the study area

**Fig. 1:**
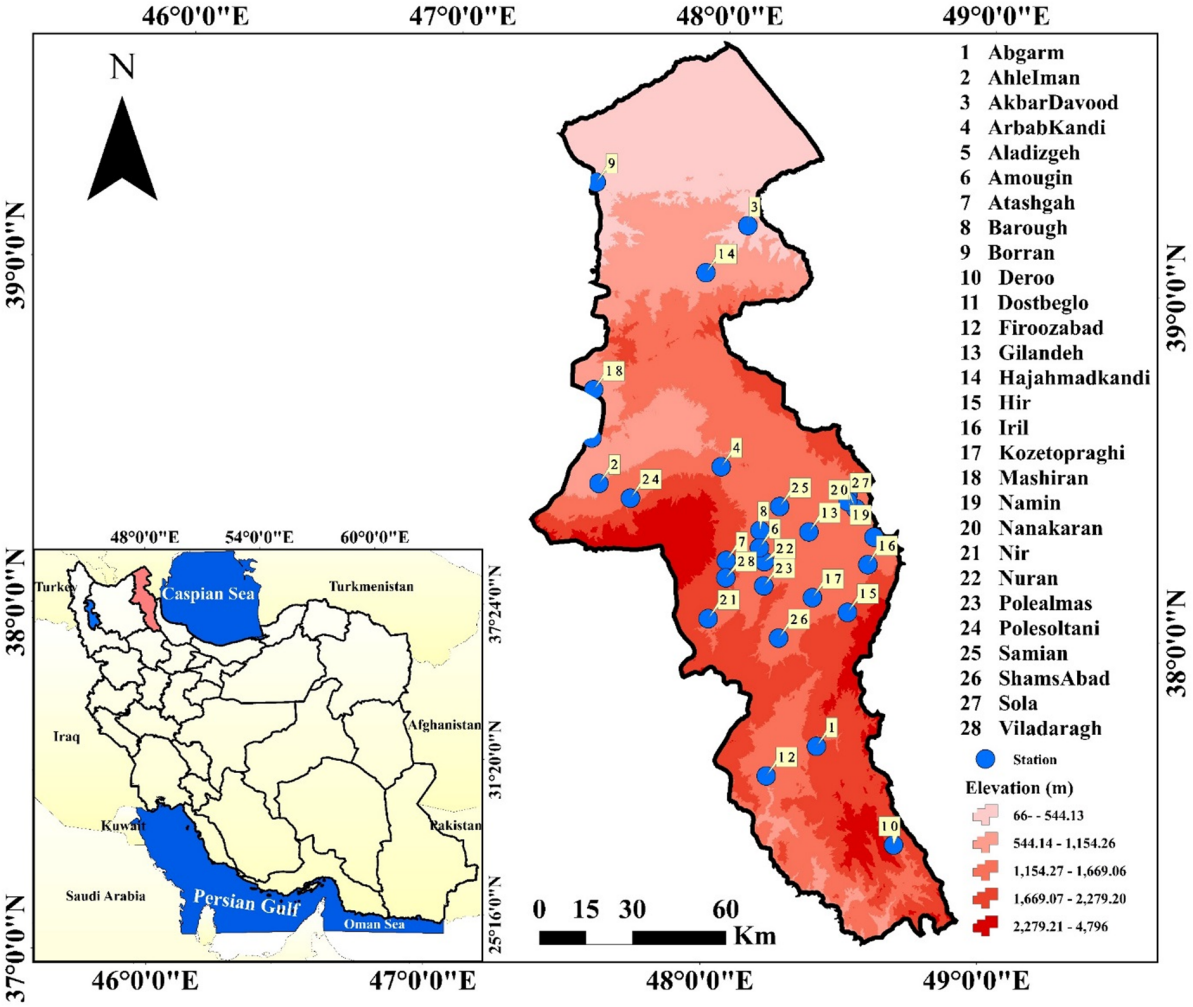
The geographic location of the studied river gauge stations in the Ardabil province and Iran (Map processing and creation were carried out by the researchers using ArcMap within ArcGIS version 10.1^36^ https://www.esri.com/en-us/arcgis/products/arcgis-desktop/overview).

Ardabil Province, located in northwest Iran near the border with the Republic of Azerbaijan, features diverse and mountainous topography. The study area includes 28 rivers in Ardabil province. Ardabil province has an area of 18,000 km^2^ in the northwest of the Iranian plateau and is located in the geographical range of latitudes from 37° 45′ to 39° to 42′ North and lengths from 47° 03′ to 48° 55′ east from the Greenwich Meridian. Ardabil province is bordered by the Republic of Azerbaijan to the North, Gilan to the East, Zanjan to the south and East Azerbaijan to the West. It is known as one of the mountainous regions of Iran and its average annual rainfall fluctuates between 250 and 600 mm^34^. The elevations of the province vary between 40 m and 4811 m, so that the highest point at the summit of Sablan, the lowest elevation in the city of Parsabad Mogan and the average elevation in the Ardabil plain are 4811, 40 and 1850 m from sea level respectively^24^. The western part of Ardabil province has the highest annual rainfall and the average rainfall in this area is estimated to be between 400 and 500 mm. The southern regions of the province, such as Khalkhal County, have about 350 mm of rainfall annually^35^. The climate is predominantly cold and mountainous, characterized by long winters and significant snowfall, with temperature and precipitation varying across the region due to changes in elevation. The soils in the area include developed soils in plains and foothills, fertile rangeland soils, and saline soils in parts of the central province. The province’s water resources consist of major rivers, numerous springs, and groundwater reservoirs in the plains. Vegetation cover and land use mainly comprise extensive rangelands, fertile agricultural lands, scattered forests in the northern areas, barren lands, as well as urban and rural settlements. In recent years, a decrease in rangeland and forest cover alongside an increase in agricultural land and settlement development has led to notable environmental consequences. Figure 1 shows the location of hydrometric stations on studied rivers over the study area.

### Methodology

#### Application of river analysis package (RAP) software

The river analysis package (RAP Version 3.0.8, https://toolkit.ewater.org.au/Tools/RAP) software is designed as a powerful tool for managing environmental flows and river data analysis. With multiple modules, the software allows river managers to effectively allocate and optimize environmental flows^37^. The RAP Version 3.0.8 software (https://toolkit.ewater.org.au/Tools/RAP) package is especially used to allocate water resources in order to preserve and improve aquatic ecosystems^38^. The tool is able to calculate accurate water flow statistics and Colwell indicators at different time periods and provide in-depth analysis of flow patterns. With the help of RAP analyses managers will be able to make better management decisions that help protect water resources and improve environmental status thus improving the quality of water and environmental management^39,40^. In this study, Colwell indices were calculated using RAP Version 3.0.8 software at monthly and seasonal scales based on daily flow data. The daily river flow data were sourced from Ardabil Regional Water Company with quality control to ensure accuracy. In this regard, the quality of the daily discharge data and the length of the statistical period were carefully checked. Since the data were daily, missing values were not reconstructed because the software used can handle missing data effectively. During data screening, stations with extensive missing data or poor-quality records were excluded to ensure robustness. The final dataset comprised stations with long, complete records, containing < 2% missing daily values. Missing entries were coded as null values (-9999), enabling RAP Version 3.0.8 software to exclude them from monthly/seasonal aggregate calculations. Since Colwell indices use categorized data (monthly/seasonal aggregates) rather than raw daily values, the absence of a few daily records minimally affects the representative values (mean, median, max, min) for each period, particularly given the long-term dataset context. Finally, stations from different parts of the study area with acceptable lengths of record were selected, and the database was finalized and prepared. Data were sorted and imported into RAP Version 3.0.8 software, which automatically handles missing values. Outliers were not removed, as flash floods and extreme events are considered natural parts of the flow regime and included in the analysis. Daily flows were aggregated into these intervals, and indices were derived from mean, median, minimum, and maximum values. This method enables detailed analysis of flow patterns under different conditions and time scales.

#### Colwell indices

Colwell indices are a measure for assessing the predictability of seasonal environmental events. This method was developed for categorized data, such as when a fruit tree bears fruit or when fish spawn. Seasons when these events occurred or did not occur are recorded, and a table of the presence or absence of various events is created. The equations developed by Colwell in 1974 can be used to predict predictability, which indicates how dependent an event is on a particular season. Constancy is a measure of the uniformity of event occurrence across all seasons, while contingency is a measure of the repeatability of seasonal patterns^39,41^. Several studies have been conducted on the use of Colwell indices in river flow analysis, focusing on quantifying the characteristics of flow regimes and evaluating the seasonality of river flows^42–44^.

Seasonality and periodicity, though often conflated, differ in Colwell’s framework: seasonality describes regular within‑year flow patterns (e.g., spring highs), while periodicity (captured by contingency) measures the repeatability of those patterns across years. In this study, we combine constancy and contingency into a single predictability metric to address both intra‑annual variation and inter‑annual recurrence in river flow regimes.

#### Calculation of predictability, constancy and contingency

Predictability is the sum of Constancy and Contingency. It represents the likelihood of predicting the occurrence of flow. When the flow is constant throughout the year (maximum Constancy) or if the pattern of high or low flow events repeats in all years (maximum Contingency), Predictability reaches its maximum^41,45^.

Constancy reaches its maximum when the flow is consistent across all seasons. For example, a desert river with multiple zero flow events would have a high Constancy value^39^.

The Contingency value reaches its maximum when the flow pattern is consistent each year. For example, if high flows repeat every winter, Contingency will reach its maximum in this case^39^. The equations used to calculate the Colwell indices are provided below^24,39^.

Let N_ij_ represent the number of times the phenomenon is observed in state *i* at time *j*. In this context, X_j_ denotes the total of column *j*, Y_i_ is the total of row *i*, Z is the overall total of all observations, t is the number of time intervals (columns), and s is the number of states (rows). Based on these definitions, predictability is calculated as follows:

1 $${X_i}=\mathop \sum \limits_{{i=1}}^{z} {N_{ij}}$$

2 $${Y_i}=\mathop \sum \limits_{{j=1}}^{t} {N_{ij}}$$

3 $$Z=\mathop \sum \limits_{i} \mathop \sum \limits_{j} {N_{ij}}=\mathop \sum \limits_{j} {X_j}=\mathop \sum \limits_{j} {Y_i}$$

4 $$H\left( X \right)= - \mathop \sum \limits_{{j=1}}^{t} \frac{{{X_j}}}{Z}\log \frac{{{X_i}}}{Z}$$

5 $$H\left( {XY} \right)= - \mathop \sum \limits_{i} \mathop \sum \limits_{J} \frac{{{N_{ij}}}}{Z}\log \frac{{{N_{ij}}}}{Z}$$

6 $$Predictability\left( P \right)=1 - \frac{{H\left( {XY} \right) - H\left( X \right)}}{{\log S}}$$

7 $$Constancy\left( C \right)=1 - \frac{{H\left( Y \right)}}{{\log S}}$$

8 $$Contingency\left( M \right)=1 - \frac{{H\left( X \right)+H\left( Y \right)+H\left( {XY} \right)}}{{\log S}}$$

H(X) represents the uncertainty over time. It reaches zero when all observations occur in only one time period (one Xj = Z, others = 0), and reaches its maximum (log t) when all time periods are equally likely (Xj = Z|t). Similarly, H(XY) expresses the uncertainty in the combined effect of time and state. The conditional uncertainty of state given time is Hx(Y) = H(XY) – H(X), which ranges from 0 to log s. By dividing by log s, it is standardized to a 0–1 scale. Since predictability is the opposite of uncertainty, it is calculated as 1 – Hx(Y). In the Colwell equations, logarithms with base 2 are used to calculate entropy and related indices. The use of this logarithmic base is emphasized in Colwell’s original work^39^ as well as in similar studies, where it is considered the standard basis for entropy computation. The Colwell indices (Predictability (P), Constancy (C), and Contingency (M)) range from 0 to 1, with higher values indicating more structured and regular flow patterns. Based on Colwell^39^, values are categorized as low (0–0.33), moderate (0.34–0.66), and high (0.67–1) to aid interpretation.

Colwell’s indices (Predictability, Constancy, and Contingency) use information theory to analyze river flow temporal patterns. Based on Shannon’s entropy, H(X) measures temporal uncertainty, and H(XY) the combined state-time uncertainty, both scaled 0–1 using log s. Predictability is decomposed into Constancy and Contingency (P = C + M), with conditional uncertainty Hx(Y) = H(XY) – H(X), allowing multi-layered uncertainty assessment. Daily flows are categorized and analyzed across temporal scales (monthly, seasonal) and flow metrics (mean, median, min, max) using matrix computations, enabling simultaneous analysis of 28 rivers.

#### The criteria for Colwell indices analysis

At any temporal scale (monthly or seasonal), Colwell indices can be calculated based on the mean, median, minimum, or maximum daily flow values. The selected flow value for each time interval (e.g., each month) is compared across all years to create a matrix of flow occurrences. These comparisons allow for the calculation of temporal patterns (predictability, constancy, and contingency) as defined by Colwell’s method. Mean, median, minimum, and maximum flows were selected for their ability to represent distinct hydrological states: mean for average conditions, median to reduce outlier effects, and min/max to capture critical drought and flood extremes, offering clear indications into flow regime behavior.

Using mean daily flow data is beneficial when the objective is to examine typical water flow conditions over a specific time period, such as a month or a season. Median daily flow data, on the other hand, divides the dataset into two equal parts, thereby reducing the influence of outliers. This approach is particularly useful for evaluating normal flow conditions without being heavily impacted by anomalous data^39^.

When the indices are calculated using minimum daily flow data, the analysis focuses on low-flow conditions, such as those occurring during drought periods. These conditions are critical as they can significantly affect aquatic life and ecosystems. In this method, the Colwell indices are computed based on the lowest recorded daily flow for each month, which serves as a representative value for that time period. This approach is well-suited for studying droughts or other extreme low-flow scenarios^39^.

Conversely, when using maximum daily flow data, the indices are derived from the highest recorded flow values within each time interval (monthly or seasonal). This method evaluates critical conditions characterized by extreme flows, which may result in flooding or trigger specific ecological activities within the river system. It focuses on the most severe environmental states and is particularly useful for analyzing rare or extreme events^39,41^. The use of mean daily flow in Colwell index calculations does not assume any specific distribution, such as normality. Since Colwell indices rely on frequency-based categorical data, the mean simply represents central flow behavior across time intervals, without requiring parametric assumptions.

#### Monthly and seasonal time periods

In time series analysis of flow data, Colwell indices can be calculated at two temporal scales: monthly and seasonal. Specifically, these indices are computed based on the division of daily flow records into monthly or seasonal intervals, depending on the user’s objectives. Monthly-scale calculations are particularly useful when the aim is to investigate monthly patterns in water flow. For instance, the analysis can begin with the first full month of the study period, or the starting month and year can be manually specified.

On the other hand, seasonal-scale calculations focus on analyzing water flow based on annual seasons rather than individual months. This approach divides a year into a few distinct seasons. For example, in tropical regions where rainy and dry seasons are well-defined, dividing the year into two seasons (rainy and dry) may be more appropriate than dividing it into 12 months. Such analysis enables the examination of water flow under specific seasonal conditions, such as during the rainy or dry seasons, offering more significant remarks for water resource management. This is particularly significant in cases where water flow is strongly influenced by seasonal changes. For example, summer-dominant flows may exhibit similar predictability to winter-dominant flows; however, their implications for water resource management can vary significantly^39,45^. The definition of seasons in this study is based on the climatic and hydrological classification of the northwestern region of Iran. The seasons were defined using regional precipitation, temperature data, and local hydrological patterns, and were approximated as the conventional climatic seasons (spring, summer, autumn, and winter). This categorization aligns with the natural flow regimes of the rivers and facilitates meaningful temporal comparisons within the study. Furthermore, the analyses were conducted on both monthly and seasonal scales to enable a more detailed and climate-relevant examination of river flow characteristics.

Table 1 presents the indices used in this study to evaluate changes in river flow regimes.

**Table 1:** Colwell indices utilized for assessing changes in river flow regimes in the present study (P = Predictability, C = Constancy, M = Contingency).

Index	Monthly time period	Index	Seasonal time period
P_MDFM	Predictability based on monthly mean daily flow	P_MDFS	Predictability based on seasonal mean daily flow
C_MDFM	Constancy based on monthly mean daily flow	C_MDFS	Constancy based on seasonal mean daily flow
M_MDFM	Contingency based on monthly mean daily flow	M_MDFS	Contingency based on seasonal mean daily flow
P_MedM	Predictability based on monthly median daily flow	P_MedS	Predictability based on seasonal median daily flow
C_MedM	Constancy based on monthly median daily flow	C_MedS	Constancy based on seasonal median daily flow
M_MedM	Contingency based on monthly median daily flow	M_MedS	Contingency based on seasonal median daily flow
P_MinM	Predictability based on minimum monthly flow	P_MinS	Predictability based on minimum seasonal flow
C_MinM	Constancy based on minimum monthly flow	C_MinS	Constancy based on minimum seasonal flow
M_MinM	Contingency based on minimum monthly flow	M_MinS	Contingency based on minimum seasonal flow
P_MaxM	Predictability based on maximum monthly flow	P_MaxS	Predictability based on maximum seasonal flow
C_MaxM	Constancy based on maximum monthly flow	C_MaxS	Constancy based on maximum seasonal flow
M_MaxM	Contingency based on maximum monthly flow	M_MaxS	Contingency based on maximum seasonal flow

**Fig. 2:**
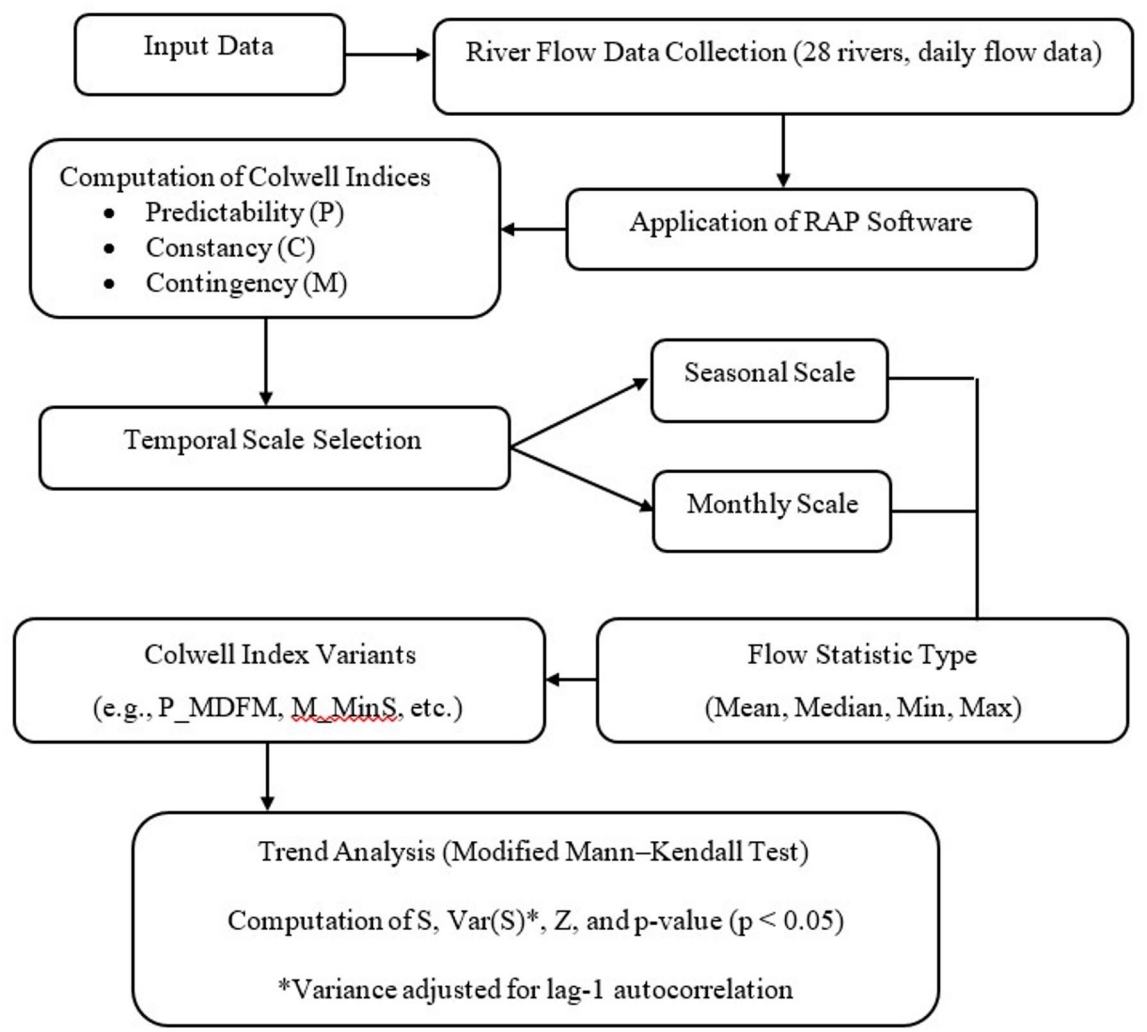
Flowchart illustrating the stages of the research process.

The stages of the research process are shown in the form of a flowchart in Fig. 2.

#### Trend detection in Colwell indices

In hydrological analysis, examining the temporal changes in river flow discharge (at daily, monthly, and annual time scales) provides valuable information about variations in flow regime components^46^. In this study, the Modified Mann–Kendall (MMK) test^47^ was applied to detect trends in streamflow (discharge) time series at multiple hydrometric stations. The MMK test was chosen because it accounts for serial correlation, which can otherwise bias the results of the classical Modified Mann–Kendall test^48,49^. To quantify the magnitude and direction of trends, the Theil–Sen slope estimator was used.

The Modified Mann-Kendall test and Theil-Sen slope estimator were implemented in R version 4.5.2 software using custom scripts (https://www.r-project.org/). For each station, the MMK statistic S was computed, and the variance was adjusted for serial correlation (lag-1). The Theil–Sen estimator was used to calculate the median slope, providing the magnitude and direction of trends. P-values and trend directions were determined for all hydrometric stations, and results were exported to CSV for further analysis.

## Results and discussion

**Fig. 3:**
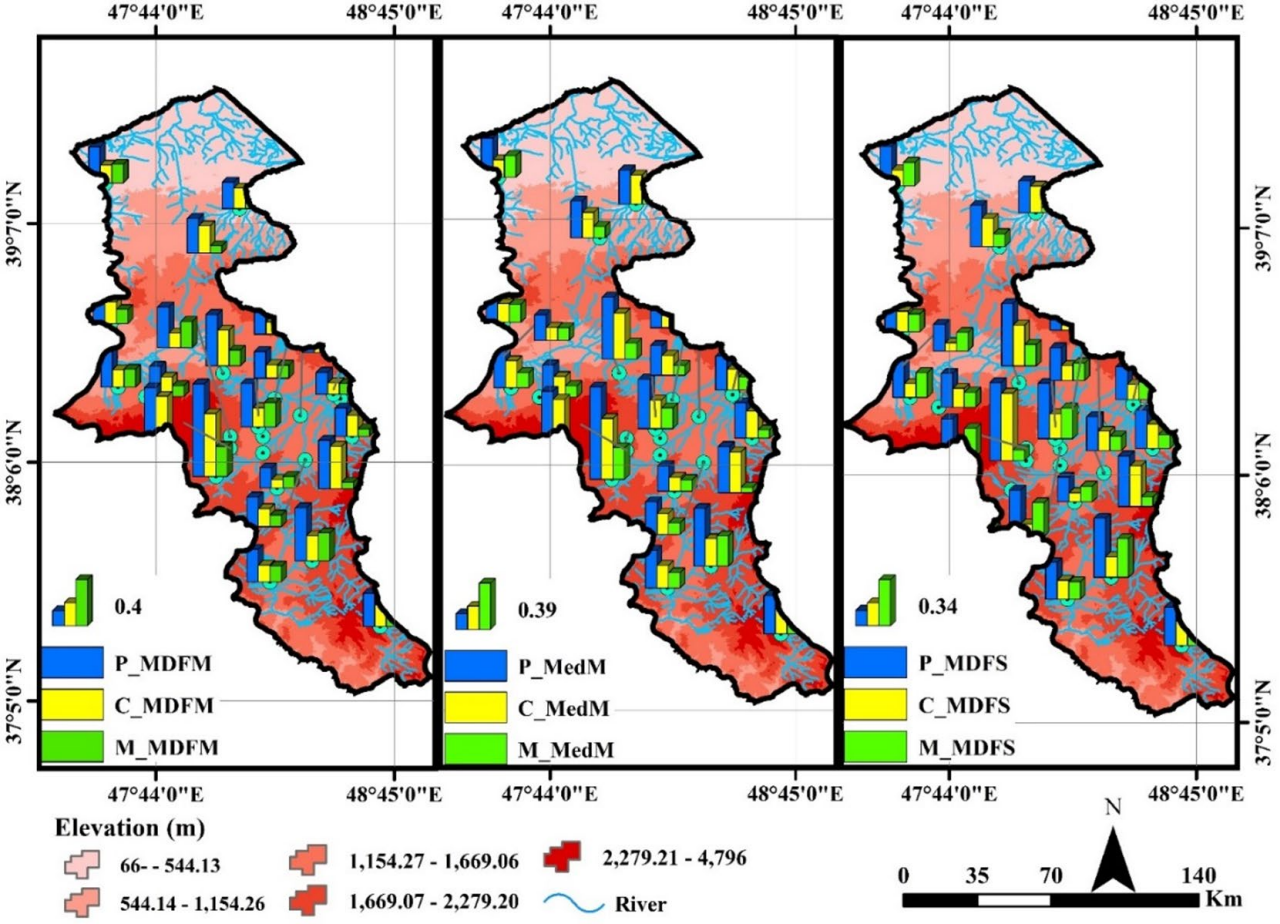
Spatial variations and comparison of Colwell indices calculated based on mean daily flow data (Map processing and creation were carried out by the researchers using ArcMap within ArcGIS version 10.1^36^ https://www.esri.com/en-us/arcgis/products/arcgis-desktop/overview).

The results of Colwell indices calculated based on the mean daily flow data (P_MDFM, C_MDFM, and M_MDFM) for the studied rivers are presented in Fig. 3.

Based on the results shown in Fig. 3, the Nir River, with the highest values for P_MDFM (0.79), C_MDFM (0.54), and M_MDFM (0.26) compared to the other rivers, is identified as having predictable and relatively stable flow patterns.

The Nir River shows varying predictability depending on the Colwell index used. It has high predictability based on monthly mean (P_MDFM = 0.79) and median flows (P_MedM = 0.78), indicating stable average conditions. However, predictability for minimum seasonal flow (P_MinS = 0.64) is slightly lower, reflecting greater variability during low-flow periods due to factors like drought or irregular climate patterns.

On the other hand, rivers like Aladizgeh, due to their lower index values (0.16 for Predictability), are recognized as a location with greater variations and lower predictability. In general, according to the results, stations such as Nir and Viladaragh exhibit the highest P_MDFM values, while rivers such as AkbarDavood and Aladizgeh show the lowest values. Rivers like Hir and Polealmas have the highest C_MDFM values, indicating relatively stable flow patterns in these rivers. High C_MDFM values in rivers like Hir and Polealmas suggest stable, uniform flows throughout the year with low intra-annual variability. This indicates minimal seasonal fluctuations, possibly due to groundwater inputs, flow regulation (Yamchi Dam), or consistent precipitation, conditions beneficial for aquatic ecosystems by reducing flow-related stress.

**Fig. 4:**
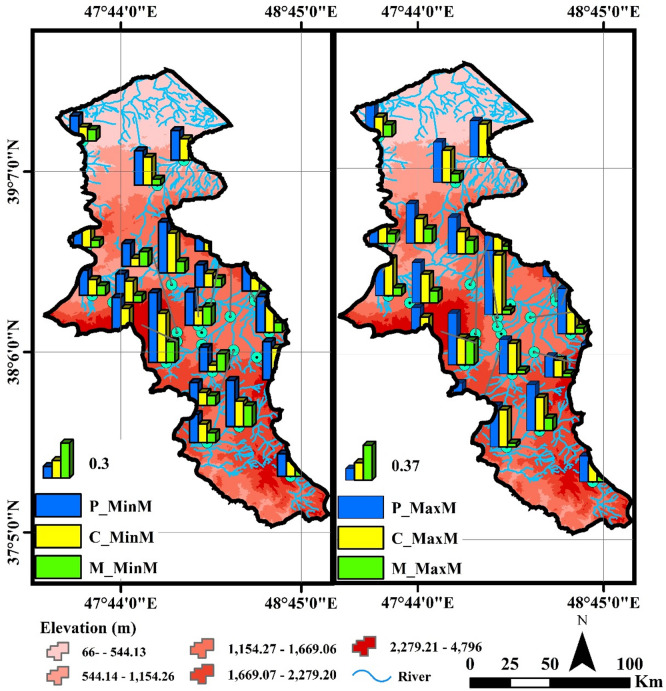
Spatial variations and comparison of Colwell indices calculated based on the minimum and maximum daily flow data (Map processing and creation were carried out by the researchers using ArcMap within ArcGIS version 10.1^36^ https://www.esri.com/en-us/arcgis/products/arcgis-desktop/overview).

Nir River has the highest M_MDFM value, indicating a high repeatability of the monthly flow pattern. These index variations may result from hydrological and geographical factors. Rivers like Nir, with higher predictability and constancy, likely benefit from stable snowmelt, consistent groundwater input, and moderate climate. In contrast, rivers like Aladizgeh may experience flashier flows due to intense rainfall, smaller basins, or rugged terrain. Figure 3 shows high P_MedM and C_MedM values for the Nir and Viladaragh rivers (0.78 & 0.60; 0.51 & 0.53), indicating smoother, more predictable monthly flow patterns. In contrast, Dostbeglo and Namin rivers have lower values (P_MedM: 0.21, 0.20; C_MedM: 0.11, 0.12), reflecting less stable flows. This variation reflects local climatic, topographic, and hydrological influences. Nir also had the highest P_MedS, C_MedS, and M_MedS values, while Namin, Dostbeglo, and AkbarDavood showed the lowest. In rivers with flashy flows (e.g., Dostbeglo, Namin), mean-based predictability was higher due to extreme events, while median-based values were lower, reflecting typical flow. In contrast, stable rivers (e.g., Nir) showed similar values for both. This contrast indicates the usefulness of median-based indices in identifying flow regime stability and distinguishing basins with variable versus consistent flow patterns. The observed differences between mean-based and median-based indices may reflect variations in the hydrological behavior of the basins. Median-based indices are generally less sensitive to extreme values and thus can better represent more stable baseflows. In contrast, mean-based indices may be influenced by high-magnitude, short-duration flow events. Therefore, rivers showing notable discrepancies between these two types of indices likely experience more variable and unstable flows, while smaller differences suggest more uniform and stable flow regimes. The P_MDFS, C_MDFS, and M_MDFS results for seasonal average daily flow reveal varied flow patterns among rivers. Kozetograghi River (P_MDFS = 0.77) shows stable, predictable flow, while ShamsAbad River (P_MDFS = 0.03) is highly variable and unpredictable. These differences reflect influences from geography, watershed, and climate. C_MDFS values range from 0.02 (Aladizgeh) to 0.53 (Viladaragh), indicating flow stability over time. The results of the spatial changes in Colwell index values at the monthly time scale (calculated based on the minimum and maximum daily flow data) are shown in Fig. 4.

According to the results presented in Fig. 4, the analysis of the P_MinM, C_MinM, and M_MinM indices based on the minimum monthly flow showed that the Nir River, with a P_MinM value of 0.60 and a C_MinM value of 0.42, exhibits the highest Predictability and Constancy among the rivers.

This indicates that the minimum monthly flows in this river are stable and predictable. The Viladaragh River, with P_MinM equal to 0.50 and C_MinM equal to 0.41, shows a similar pattern. In contrast, rivers such as Aladizgeh, Namin, and Dostbeglo, with lower P_MinM and C_MinM values, exhibit lower Predictability and Constancy in their minimum monthly flows. The analysis of the P_MaxM, C_MaxM, and M_MaxM indices based on the maximum monthly flow revealed that the ArbabKandi River, with a P_MaxM value of 0.74, and the Nuran River, with a P_MaxM value of 0.70, have the highest levels of Predictability and Constancy for maximum monthly flows. This suggests that the maximum monthly flows in these rivers occur consistently and predictably. In contrast, rivers such as Hir and Deroo show lower values of Predictability and Constancy, indicating greater variability and less predictability in the maximum monthly flows in these regions.

**Fig. 5:**
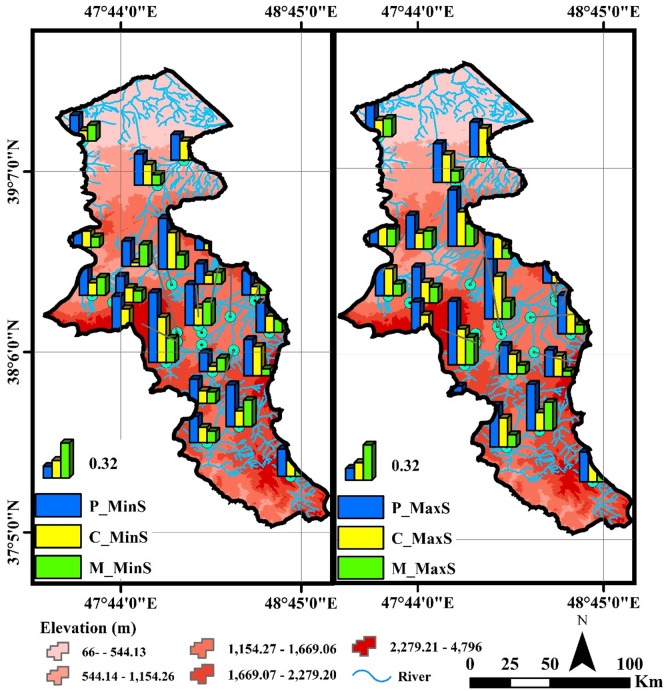
Spatial variations and comparison of seasonal Colwell indices calculated based on the minimum and maximum daily flow data (Map processing and creation were carried out by the researchers using ArcMap within ArcGIS version 10.1^36^ https://www.esri.com/en-us/arcgis/products/arcgis-desktop/overview).

The spatial variations and comparison of the Colwell index values at the seasonal time scale (calculated based on the minimum and maximum daily flow data) are shown in Fig. 5.

According to the results presented in Fig. 5, the analysis of the P_MinS, C_MinS, and M_MinS indices based on the minimum seasonal flow in the rivers of Ardabil province shows significant differences in the characteristics of the minimum flows. The Nir River, with the highest values of P_MinS (0.64), C_MinS (0.42), and M_MinS (0.23), exhibits a predictable, stable flow pattern with high contingency for minimum seasonal flows. The results indicate that the minimum flows in this river occur consistently and predictably. Similarly, the Viladaragh River, with high values of P_MinS (0.54) and C_MinS (0.42), also demonstrates predictable and stable flows, but the lower M_MinS value (0.12) suggests a lower level of contingency. In Viladaragh, high predictability and constancy combined with low contingency indicate a relatively stable flow throughout the year. This pattern may result from steady snow-fed regime of the river discharges. While the flow remains consistent (high constancy), the lack of distinct seasonal peaks leads to low contingency. In contrast, rivers such as Atashgah and Dostbeglo, with lower values (P_MinS = 0.23, 0.13 and C_MinS = 0.03, 0.03), show minimum flows with reduced predictability and constancy, indicating high variability and irregularity in the minimum flows of these rivers. In rivers like Atashgah, variability is caused by steep topography, rapid hydrological responses, sudden rainfall, and rapid snowmelt, resulting in unstable and unpredictable flows. In contrast, rivers such as Nir and ArbabKandi, with more stable natural conditions and minimal human interventions, exhibit more regular and predictable flows. These factors explain the variability of flow indices across different regions. The P_MaxS, C_MaxS, and M_MaxS indices reveal distinct flow behaviors among Ardabil’s rivers. ArbabKandi and Nir show high, stable, and predictable seasonal flows, while Hir and Namin exhibit lower, less stable flows, reflecting climatic and topographic influences. These results align with Yang et al. (2019) and Poff et al. (2002), who link climate change and variable rainfall to reduced flow predictability, especially in less stable rivers like Hir. Thompson et al. (2011) demonstrated that effective water management can enhance flow stability in arid regions, consistent with observations in ArbabKandi and Nir. Olden and Poff (2003) noted that flow variability impacts aquatic ecosystems, matching patterns seen here. Arthington et al. (2006) emphasized managing environmental flows to sustain river ecosystems, supporting the need for tailored flow management in this study area.

High Predictability values in the Nir River (0.79) and the Arbabcandi River (0.74) indicate strong repeatability of the flow pattern throughout the year, suggesting that these rivers are governed by hydrological systems with long-term memory. The results showed that predictability in these basins appears to be influenced by groundwater and snowmelt patterns. In contrast, the P_MDFM value in the Aladizgeh River (0.16) is very low, indicating a lack of flow repetition and a rapid response of this river to short-term rainfall events and direct rainfall–runoff. This behavior reflects the steep slope of the basin, which leads to unstable and unpredictable flows.

Regarding Constancy, the C_MinS value of 0.42 for the Nir River indicates that the seasonal minimum flows do not fluctuate significantly, which is due to the persistence of baseflow during summer. In the Dostbeglo River, the C_MinS value is only 0.03; this low value results from riverbed drying in summer, excessive water withdrawal for agriculture, and the absence of hydrological storage upstream.

As for the interannual repeatability of seasonal flow patterns (Contingency), the M_MaxS value of 0.21 in the Arbabcandi River shows that peak flows tend to occur in nearly the same season (spring). In the Hir River, the M_MaxS value is less than 0.05, meaning the timing of peak flow changes from year to year. This may be due to the impact of the dam located upstream, constructed downstream of Lake Neur, which artificially regulates the flow and alters the natural flood occurrence cycle.

**Fig. 6:**
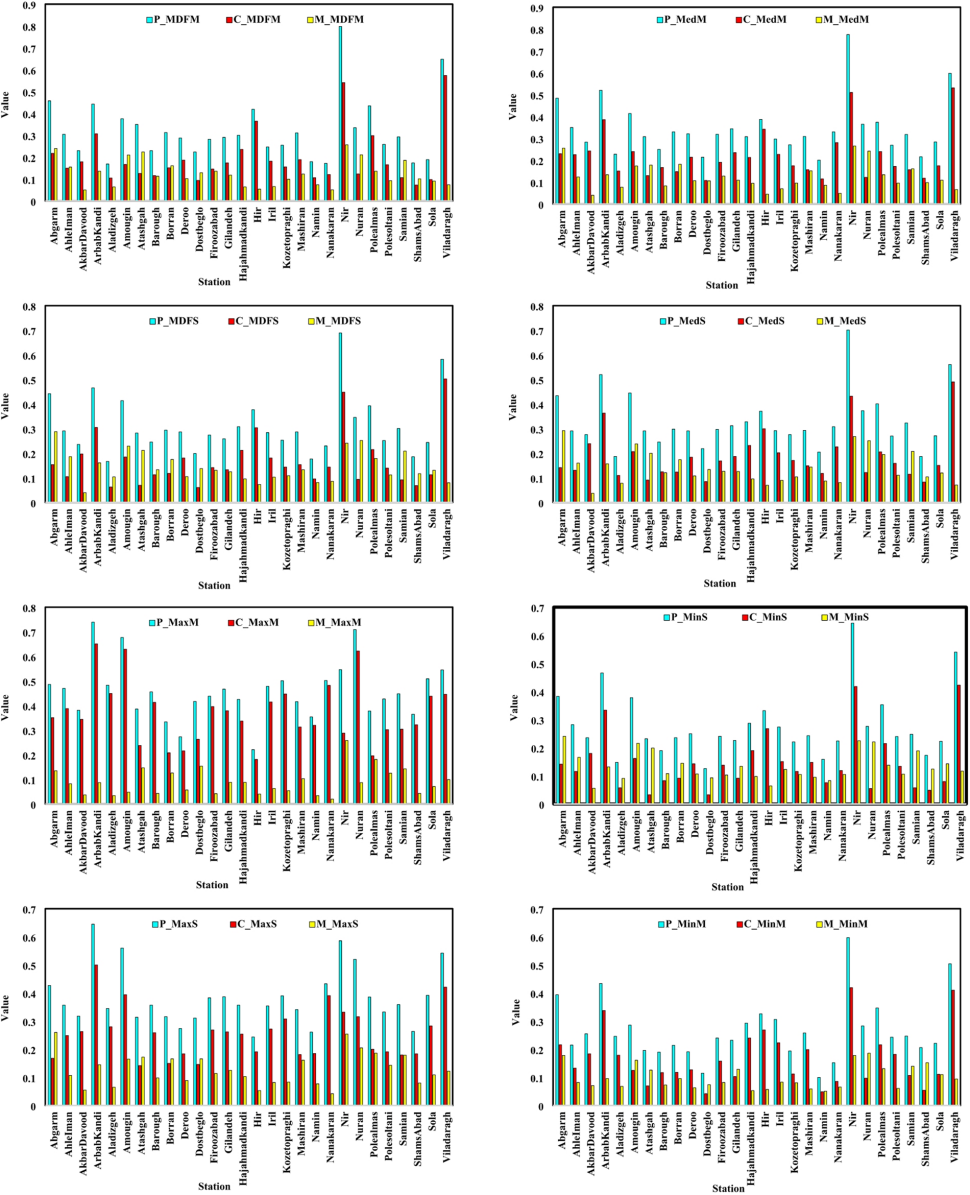
Comparison of Colwell Indices of Flow Discharge in Rivers of Ardabil Province.

The comparison results of the Colwell indices for river flows in the studied rivers are presented in Fig. 6.

According to the results presented in Fig. 6, it can be observed that the Nir and Viladaragh rivers exhibit higher values across various indices, particularly for Predictability and Constancy. The high predictability and stability of the Nir River’s flow regime (e.g., P_MinS = 0.64, C_MinS = 0.42) result from several hydrological and geographical factors. Snowmelt from moderate to high elevations provides a regular seasonal runoff source, while groundwater-fed baseflow maintains streamflow during dry periods. Downstream aquifers, especially near cultivated orchards, act as buffers, enhancing low-flow constancy. Stable land cover, including rangelands and forests, reduces runoff variability and promotes infiltration. Moreover, limited human intervention in the upper and middle basin helps preserve the natural flow regime, supporting higher predictability, particularly under minimum flow conditions.

The ArbabKandi and Amougin rivers also demonstrate high values for Constancy and Predictability, indicating significant seasonal flow variations. On the other hand, the Nanakaran river shows lower values for most indices, which may suggest more stable river flows with fewer fluctuations in this region.

**Fig. 7:**
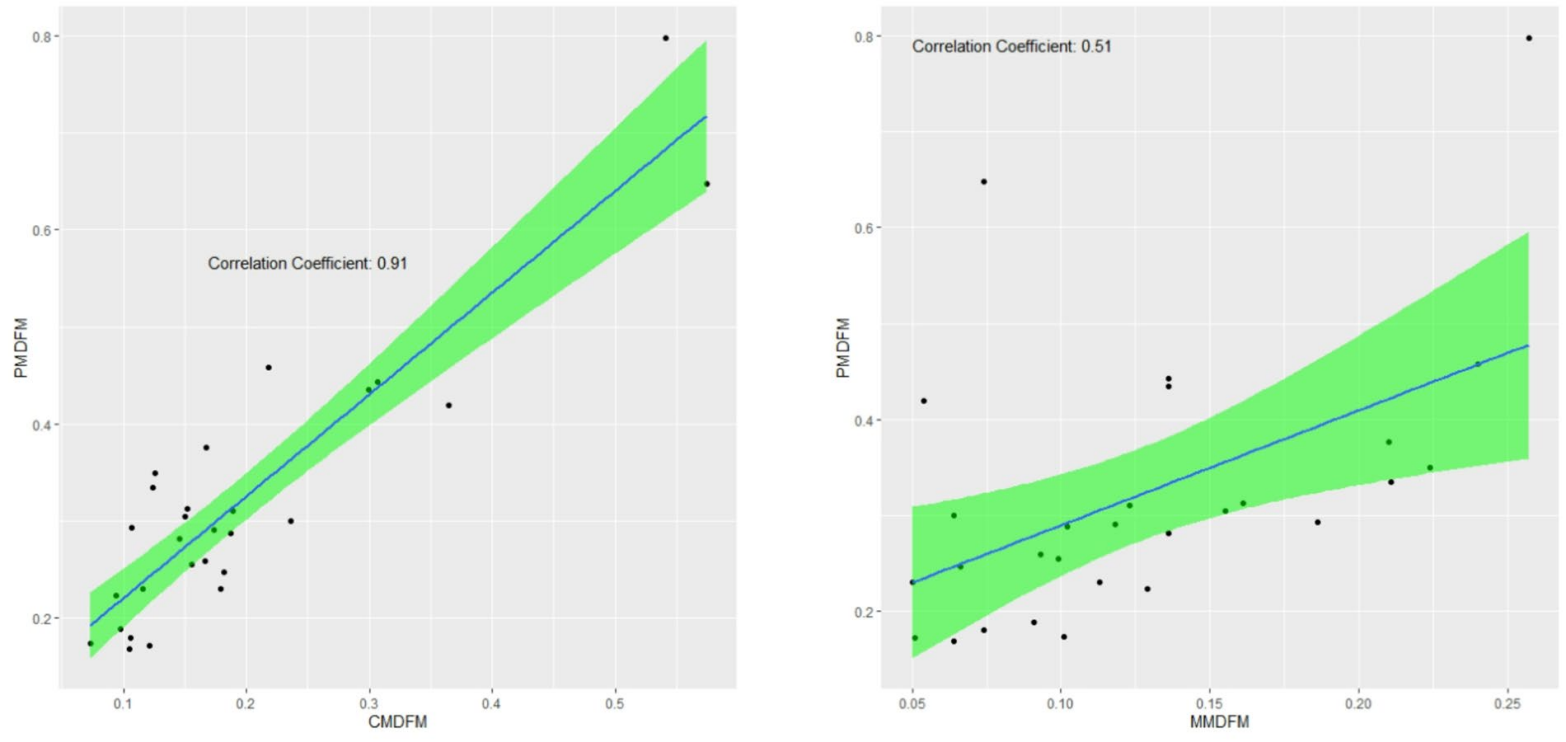
Correlation of values between colwell indices calculated based on average daily flow.

The correlation between the Colwell indices calculated based on the average daily flow is shown in Fig. 7.

According to the results, the analysis of the relationship between the P_MDFM, C_MDFM, and M_MDFM indices revealed that the correlation between P_MDFM and C_MDFM is 91%, while the correlation between P_MDFM and M_MDFM is 51%. The strong correlation (91%) between predictability (P_MDFM) and constancy (C_MDFM) suggests that predictable flows often coincide with stable, regular patterns, likely driven by steady sources like snowmelt or groundwater. In contrast, the moderate correlation (51%) between predictability and contingency (M_MDFM) indicates that while timing patterns matter, they are more affected by local factors like seasonal shifts or specific hydrologic events.

Correlation analysis showed a very strong relationship (91%) between Predictability based on monthly mean daily flow and Constancy based on monthly mean daily flow, indicating the influence of continuous processes such as baseflow in determining the stability of the flow regime. Temporal variations on monthly and seasonal scales showed that monthly indices exhibit, on average, 15% higher variability than seasonal indices. This difference is mainly due to the sensitivity of the monthly scale to extreme events.

**Fig. 8:**
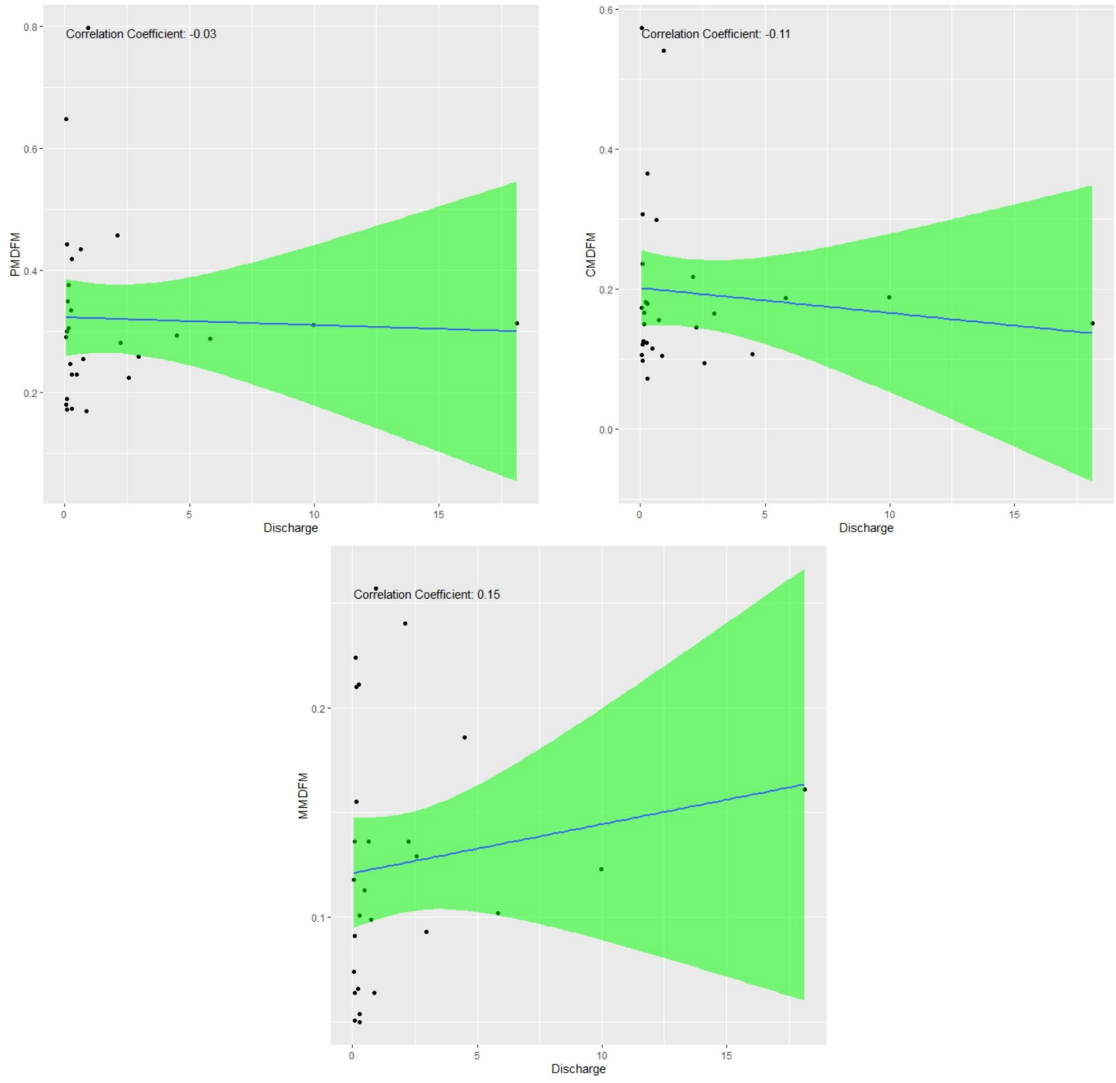
Correlation between flow values (m³/s) and Colwell indices calculated based on average daily flow.

The correlation between flow values (m^3^/s) and the Colwell indices calculated based on the average daily flow is shown in Fig. 8.

According to Fig. 8, scatter plots show a generally weak correlation between Colwell’s indices (P, C, M) and mean daily discharge. This suggests that discharge magnitude has little effect on the predictability or stability of monthly flow variations, indicating river flow dynamics are mostly independent of mean daily discharge within the observed range.

**Fig. 9:**
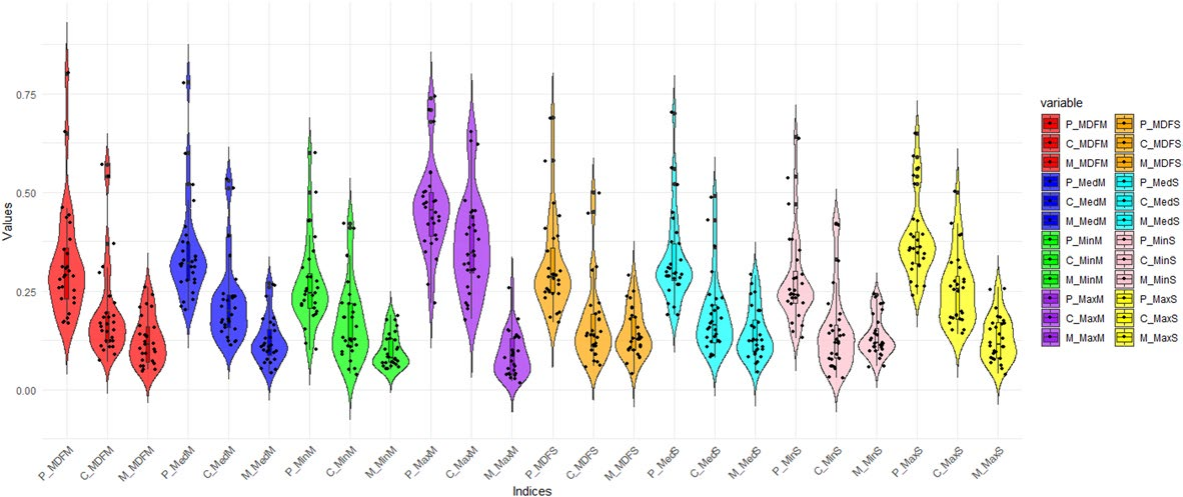
Violin plot of Colwell’s indices, including Predictability (P), Constancy (C), and Contingency (M), based on mean, median, minimum, and maximum flow at monthly and seasonal time scales.

The distribution of Colwell indices values on a monthly and seasonal scale in the rivers of Ardabil Province is shown in Fig. 9 using violin plots.

Figure 9 shows distinct patterns in the distribution of Colwell indices, indicating variations in river flow characteristics across different time periods and metrics. Monthly indices (MDFM) exhibit more variability than seasonal indices (MDFS), indicating that monthly flows are more dynamic. Predictability (P) indices tend to be lower than constancy (C) and contingency (M) indices, suggesting that flow regimes are less predictable. Among predictability indices, those based on minimum flows (MinM, MinS) show the lowest values, indicating that minimum flows are the least predictable. Minimum river flows are less predictable because they are influenced by variable factors like droughts, evaporation, and human withdrawals. These cause greater fluctuations and irregular patterns, making minimum flows harder to forecast than average or maximum flows.

Indices based on maximum flows (MaxM, MaxS) generally show higher values than those based on minimum flows, suggesting maximum flows are more consistent and predictable. Maximum flows are more predictable because they usually result from regular seasonal events like snowmelt or spring/autumn rainfalls in the study area, making their timing and size more consistent than low or average flows.

Median-based indices (MedM, MedS) fall between mean and minimum-based indices in terms of distribution. These results emphasize the need to consider both temporal scale (monthly vs. seasonal) and flow metric (mean, median, maximum, minimum) when analyzing flow regimes. Understanding these variations provides valuable perspectives into the ecological and hydrological processes influenced by river flow dynamics.

Descriptive statistics of various predictability (P), constancy (C), and contingency (M) indices at monthly and seasonal time scales are presented in Table 2.

**Table 2:** Descriptive statistics of predictability (P), constancy (C), and contingency (M) indices at monthly and seasonal time scales.

Scale	Statistics	P_MDFM	C_MDFM	M_MDFM	P_MedM	C_MedM	M_MedM	P_MinM	C_MinM	M_MinM	P_MaxM	C_MaxM	M_MaxM
Monthly	Mean	0.32	0.19	0.13	0.35	0.22	0.12	0.27	0.17	0.10	0.46	0.37	0.09
	SE	0.03	0.02	0.01	0.02	0.02	0.01	0.02	0.02	0.01	0.02	0.02	0.01
	Med.	0.29	0.16	0.12	0.32	0.20	0.11	0.24	0.13	0.08	0.45	0.35	0.08
	SD	0.14	0.12	0.06	0.12	0.11	0.06	0.11	0.10	0.04	0.12	0.12	0.05
	Kurt.	4.25	4.10	-0.43	4.78	2.93	0.50	2.36	1.16	-0.62	1.12	0.29	1.96
	Skew.	1.85	2.05	0.72	1.96	1.72	0.96	1.35	1.20	0.80	0.64	0.71	1.26
	Min	0.17	0.07	0.05	0.20	0.11	0.04	0.10	0.04	0.05	0.22	0.18	0.02
	Max	0.80	0.57	0.26	0.78	0.53	0.27	0.60	0.42	0.19	0.74	0.65	0.26

Scale	Statistics	P_MDFS	C_MDFS	M_MDFS	P_MedS	C_MedS	M_MedS	P_MinS	C_MinS	M_MinS	P_MaxS	C_MaxS	M_MaxS
Seasonal	Mean	0.31	0.17	0.14	0.33	0.19	0.14	0.28	0.15	0.13	0.38	0.26	0.13
	SE	0.02	0.02	0.01	0.02	0.02	0.01	0.02	0.02	0.01	0.02	0.02	0.01
	Med.	0.29	0.14	0.13	0.30	0.16	0.12	0.24	0.13	0.12	0.36	0.26	0.12
	SD	0.12	0.11	0.06	0.12	0.10	0.07	0.12	0.10	0.05	0.10	0.09	0.06
	Kurt.	2.99	3.62	-0.17	3.06	2.71	-0.11	2.98	2.04	-0.27	0.70	0.63	-0.06
	Skew.	1.62	1.87	0.69	1.60	1.68	0.79	1.63	1.53	0.79	1.09	0.97	0.62
	Min	0.17	0.06	0.04	0.19	0.08	0.04	0.13	0.03	0.06	0.24	0.14	0.04
	Max	0.69	0.50	0.29	0.70	0.49	0.29	0.64	0.42	0.24	0.65	0.50	0.26

According to the descriptive statistics in Table 2, predictability (P) and constancy (C) values are higher than contingency (M) values in both scales, indicating that flow patterns tend to be more regular and stable than temporally contingent. Notably, monthly predictability based on maximum flow (P_MaxM) shows the highest mean value (0.46), compared to its seasonal counterpart (P_MaxS: 0.38), suggesting greater predictability in maximum flows at shorter time scales. Differences between monthly and seasonal scales are also evident in the variability of indices. Standard deviations for monthly predictability indices such as P_MDFM (0.14) are somewhat larger than seasonal ones like P_MDFS (0.12), implying higher variability in monthly flow predictability. Additionally, kurtosis and skewness values reveal more pronounced asymmetry and peakedness in monthly data (e.g., Kurtosis of 4.25 for P_MDFM vs. 2.99 for P_MDFS), which may reflect more extreme or irregular flow events at finer temporal resolution. In conclusion, the table indicates that river flow regimes exhibit greater predictability and constancy at monthly scales than at seasonal scales, but also greater variability and extremeness in their statistical distribution. To better assess variability in predictability and constancy, statistical tools like the Coefficient of Variation (CV) and boxplots are helpful. They reveal data dispersion and indicate rivers with higher CVs as having more fluctuating and unstable flows.

Descriptive statistics of Colwell indices reveal that predictability (P) and constancy (C) are consistently higher than contingency (M), indicating that river flows in northwestern Iran are generally stable and regular rather than temporally contingent. Monthly indices, particularly for maximum flows (P_MaxM), show higher means and greater variability compared to seasonal indices, suggesting that short-term flow extremes are more predictable but also more variable. Skewness and kurtosis patterns indicate that monthly flows exhibit occasional extreme events, whereas seasonal indices smooth these variations. Overall, Table 2 indicate that flow predictability and stability differ by temporal scale and flow metric, reflecting the influence of local hydrology and climate on river regime dynamics.

**Fig. 10:**
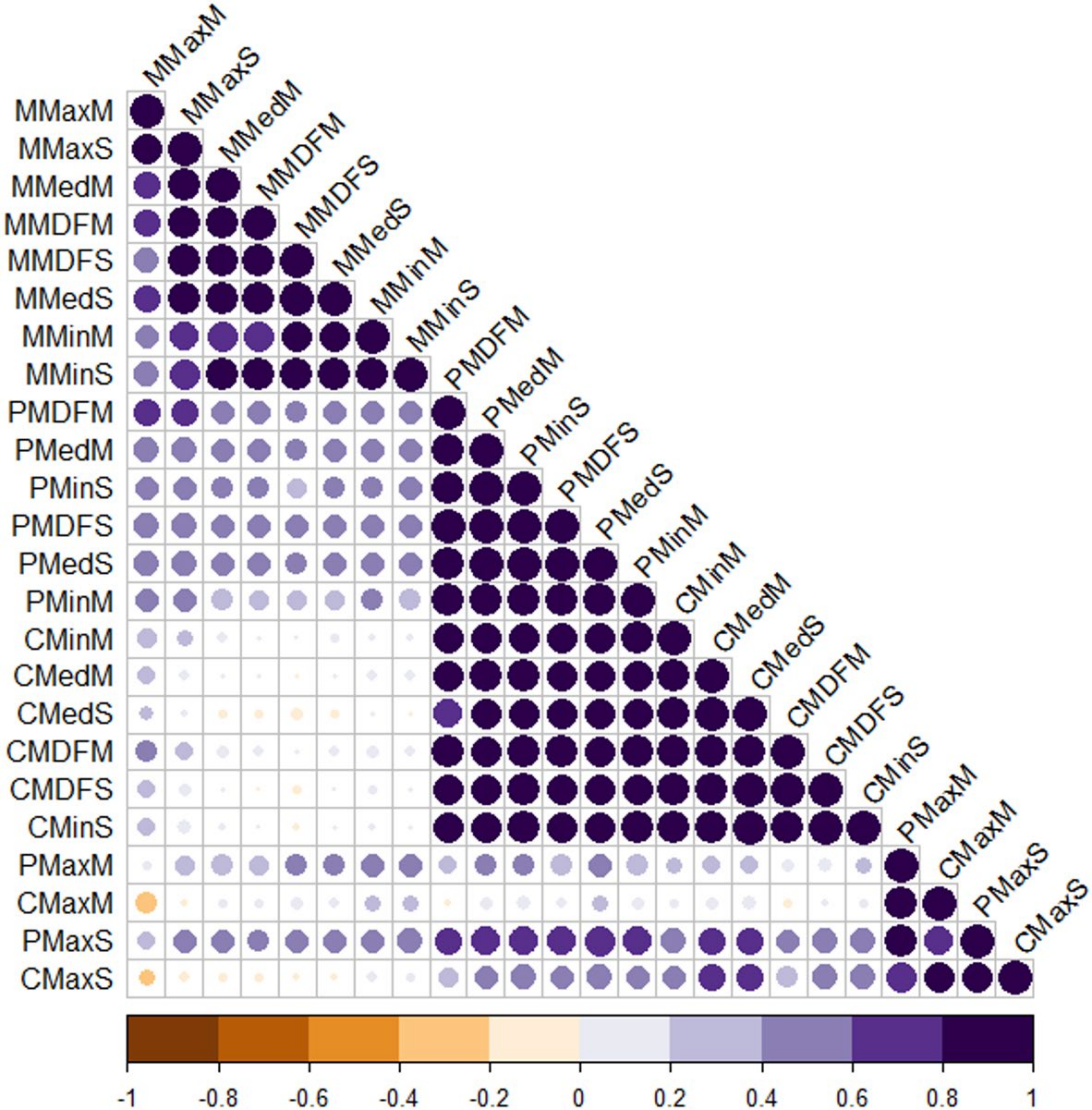
Correlation between Colwell indices of river flow.

The correlation between Colwell indices of river flow is presented through a heat map in Fig. 10.

According to the results presented in Fig. 10, the correlation matrix of Colwell indices indicates strong and significant relationships between several similar indices. Indices such as M-MaxM and M-MaxS, as well as P-MDFM, C-MDFM, and M-MDFM, exhibit a high positive correlation with one another. This suggests that these indices tend to change simultaneously and follow similar patterns.

**Fig. 11:**
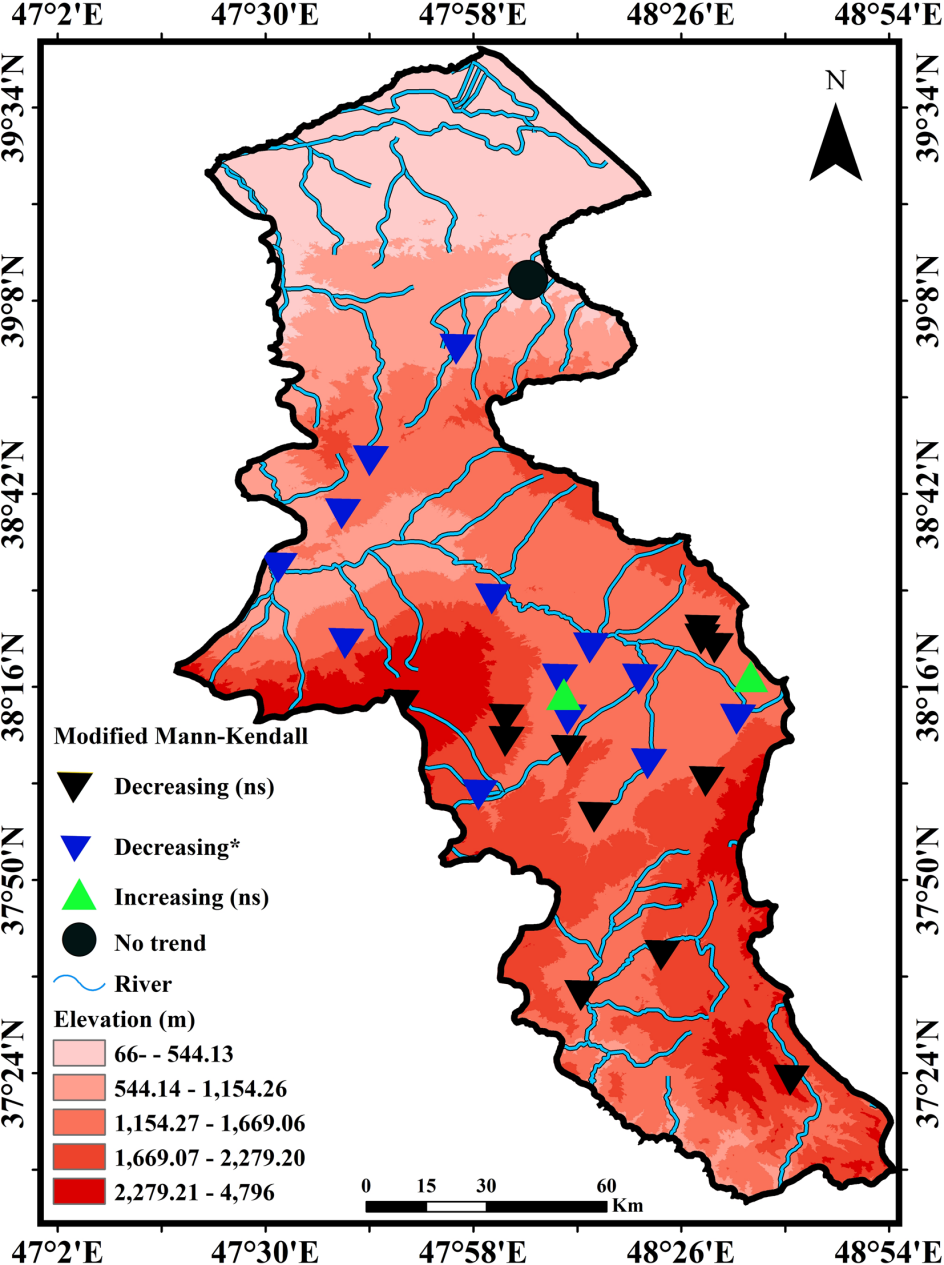
and Table 3 presents the temporal trend of the Colwell index using the Modified Mann-Kendall test for rivers in Ardabil Province.

Figure 11 Temporal trend of annual mean discharge values based on the Modified Mann-Kendall test in the study area (Map processing and creation were carried out by the researchers using ArcMap within ArcGIS version 10.1^36^ https://www.esri.com/en-us/arcgis/products/arcgis-desktop/overview).

**Table 3:** Temporal trend of river flow values using modified Mann-Kendall test at rivers of ardabil province.

Station	MMK-test value	VarS_Mod	Z	p_value	Trend	Significance	Slope
Abgarm	-17	814.89	-0.60	0.55	Decreasing	ns	-0.03
AhleIman	-7	109.27	-0.67	0.50	Decreasing	ns	-0.01
AkbarDavood	0	2377.70	0.00	1.00	No trend	ns	0.00
Arbabkandi	-75	1255.40	-2.12	0.03	Decreasing	*	-0.17
Aladizgeh	3	284.95	0.18	0.86	Increasing	ns	0.00
Amughin	4	459.48	0.19	0.85	Increasing	ns	0.00
Atashgah	-1	329.60	-0.06	0.96	Decreasing	ns	0.00
Barough	-79	1087.47	-2.40	0.02	Decreasing	*	0.00
Borran	-639	45336.57	-3.00	0.00	Decreasing	*	-0.42
Deroo	-114	5219.02	-1.58	0.11	Decreasing	ns	-0.02
Dostbeglo	-643	39840.45	-3.22	0.00	Decreasing	*	-0.27
Firoozabad	-37	1153.68	-1.09	0.28	Decreasing	ns	-0.09
Gilandeh	-621	68888.23	-2.37	0.02	Decreasing	*	-0.10
Hajahmadkandi	-35	319.59	-1.96	0.05	Decreasing	*	-0.01
Hir	-22	315.06	-1.24	0.22	Decreasing	ns	-0.01
Iril	-100	1671.96	-2.45	0.01	Decreasing	*	-0.01
Kozetopraghi	-450	26316.87	-2.77	0.01	Decreasing	*	-0.04
Mashiran	-665	49861.21	-2.98	0.00	Decreasing	*	-0.30
Namin	-40	893.22	-1.34	0.18	Decreasing	ns	0.00
Nanakaran	-25	520.45	-1.10	0.27	Decreasing	ns	0.00
Nir	-463	25302.79	-2.91	0.00	Decreasing	*	-0.01
Nuran	-32	235.29	-2.09	0.04	Decreasing	*	-0.01
Polealmas	-151	6350.55	-1.90	0.06	Decreasing	ns	-0.03
Polesoltani	-352	13045.20	-3.08	0.00	Decreasing	*	-0.01
Samian	-649	25420.19	-4.07	0.00	Decreasing	*	-0.24
ShamsAbad	-27	358.03	-1.43	0.15	Decreasing	ns	0.00
Sola	-78	2431.01	-1.58	0.11	Decreasing	ns	0.00
Viladaragh	-147	3347.78	-2.54	0.01	Decreasing	*	0.00

According to Table 3; Fig. 11, the Modified Mann-Kendall test shows significant decreasing trends in annual river flow at several stations in Ardabil Province. In total, 29 stations were analyzed, and 15 stations showed significant decreasing trends in annual river flow (*p* < 0.05). Significant decreasing trends were detected at stations such as Arbabkandi, Barough, Borran, Dostbeglo, Gilandeh, Hajahmadkandi, Iril, Kozetopraghi, Mashiran, Nir, Nuran, Polesoltani, Samian, Viladaragh and others (*p* < 0.05). Among these, stations like Samian (S = -649), Borran (S = -639), Dostbeglo (S = -643), and Mashiran (S = -665) show the strongest decreasing trends with very low p-values and high negative Mann-Kendall values. In contrast, stations such as Abgarm (*p* = 0.55), AhleIman (*p* = 0.50), Hir (*p* = 0.22), ShamsAbad (*p* = 0.15), Namin (*p* = 0.18), Nanakaran (*p* = 0.27) and several others exhibited no significant trends (ns), indicating relatively stable annual flows. Some stations show moderate but significant decreases at the 95% confidence level (*p* < 0.05), including Arbabkandi (S=-75), Barough (S=-79), Gilandeh (S=-621), Hajahmadkandi (S=-35), and Nuran (S=-32). Stronger decreases at the 99% confidence level (*p* < 0.01) are observed at Borran, Dostbeglo, Kozetopraghi, Mashiran, Polesoltani, Samian, and Viladaragh. The high negative S values and Sen’s slope (e.g., Borran slope=-0.42, Mashiran slope=-0.30, Samian slope=-0.24) indicate strong and consistent decreasing flow trends over time. These reductions are likely influenced by anthropogenic impacts, especially the construction of Yamchi Dam, water withdrawals for agriculture, and land-use changes in upstream watersheds. In terms of river flow trend classification, rivers fall into two groups: Significant decreasing trends (Modified MK, *p* < 0.05): Arbabkandi, Barough, Borran, Dostbeglo, Gilandeh, Hajahmadkandi, Iril, Kozetopraghi, Mashiran, Nir, Nuran, Polesoltani, Samian, Viladaragh. Non-significant or stable trends (*p* > 0.05): Abgarm, AhleIman, AkbarDavood, Aladizgeh, Amughin, Atashgah, Deroo, Firoozabad, Hir, Mashiran, Namin, Nanakaran, ShamsAbad, Sola, Polealmas. Our analysis using the Modified Mann-Kendall test uniquely improves the accuracy of trend detection by reducing the influence of serial correlation, providing more reliable evidences into hydrological changes. It confirms that approximately half of the stations still exhibit significant decreasing trends, reinforcing concerns about declining river flow due to climate variability and human activities.

The Modified Mann-Kendall test results show significant decreasing trends in annual river flow at approximately half of the studied stations, with the strongest declines observed at Polealmas (-151), Gilandeh (-556), and Borran (-476). Conversely, several rivers, such as Abgarm and Hir, exhibit no significant trends, reflecting stable hydrological conditions. The variability in trend magnitudes across stations indicates the combined influence of human interventions, such as dams and water extraction, and regional climatic factors. As a conclusion, the Table 3 emphasizes the spatial heterogeneity of river flow trends and identifies rivers most vulnerable to hydrological changes.

There is substantial evidence from multiple studies on the influence of climatic and human factors on river flow changes in Ardabil Province. For example, Mehri et al.^34^ showed that the base flow index (BFI) has a decreasing trend at most stations in Ardabil, and this decline cannot be explained by reduced precipitation alone; water extraction by humans clearly exacerbates the flow reduction. They reported a weak correlation between precipitation and base flow, indicating that human activities (agricultural water use and flow regulation) are the main drivers of decreased water yield. In Mostafazadeh & Mehri^49^, trend and flood coefficient analyses at 22 stations revealed a significant increasing trend in flood coefficients in many rivers, attributed to both increased precipitation anomalies (climatic factors) and land-use changes and rangeland degradation (human factors). In Mostafazadeh & Azizi^53^, changes in flow and sediment regimes upstream and downstream of the Yamchi and Sabalan dams were examined using quantitative indices such as annual mean flow, flow duration, and flow and sediment variability indicators, showing that dam construction led to reduced discharge, altered flow seasonality, and lower sediment load, a clear example of attributing changes to human activity. In Esfandyari Darabad et al.^54^, satellite image analysis indicated that a 20% reduction in forest cover and a 20% increase in agricultural land increased flood peaks (34.46 m³/s) and reduced time of concentration, demonstrating a direct and quantifiable relationship between land-use changes and river flow alterations. Khorooshi et al.^55^ reported a significant decline in the Hydrologic River Health Index at 13 stations, linking this deterioration to both intensified human water use and precipitation reductions due to climate change. In Ardabil Province, previous studies such as Asgari et al.^56^, using the GR4J model and climate scenarios, have shown that changes in temperature and precipitation reduce annual water yield and increase flood events, with a notable overall decline in water availability projected in future periods.

### Implication

The results consider the variability of Colwell indices in assessing river flow predictability, constancy, and contingency, essential for water resource analysis in northwestern Iran. Managers should focus on rivers like Nir and ArbabKandi for stable flow patterns, while addressing variability in rivers such as Hir and Deroo through tailored strategies that consider climatic and human factors. This approach employs Colwell indices derived from multiple flow metrics to effectively capture high flow variability and flashiness in river basins. By assessing predictability, constancy, and contingency at monthly and seasonal scales, it reflects both consistent seasonal patterns and extreme events such as floods and droughts. Determining minimum and maximum flows allows for detailed characterization of low-flow drought periods and peak flow flashiness.

The high variability of peak runoff in rivers of northwestern Iran is mainly due to intense short-duration rainfall, small watershed areas, and steep terrain slopes. Our study focused primarily on analyzing the flow regime using Colwell indices, but incorporating physical watershed characteristics could provide a better understanding of flow changes. Future research is needed to examine the correlation between flow indices and factors such as rainfall intensity, watershed size, and slope, which can provide valuable understanding. While IHA and RVA are widely used in ecohydrology, they require long-term continuous daily data and focus more on ecological flow restoration. In this study, the goal was to detect changes in flow predictability and seasonal timing across several basins with uneven data availability. Colwell indices are less sensitive to data gaps, can be calculated from categorized flow data and are computationally efficient for multi-basin comparison. Rivers with low predictability and contingency (e.g., Dostbeglo) show a loss of seasonal structure, serving as an early warning for reservoir reoperation such as the Sabalan Dam. Therefore, Colwell metrics provide a fast decision-support tool for prioritizing rivers where hydrological seasonality is being degraded.

The quantified results of this study provide practical and actionable views for water resource management. For instance, the high predictability and constancy of stable rivers such as Nir and Arbabkandi indicate a reliable water supply. This is crucial for designing long-term agricultural irrigation plans and ensuring sustainable environmental flow requirements. In contrast, rivers with low predictability and unstable hydrological behavior, such as Dostbeglo, require adaptive management strategies. These may include constructing additional storage structures or optimizing reservoir operations (e.g., at Yamchi Dam) to capture winter floods and gradually release water during dry periods to mitigate drought impacts. Moreover, the significant declining trends in flow predictability at many stations serve as an early warning for dam operators to revise storage and allocation policies and urge policymakers to reconsider water distribution plans in response to climate change and increasing human water use. By identifying rivers with high or low predictability and stability, water managers can prioritize monitoring, allocate water resources more effectively during critical low- or high-flow periods, and design location-specific strategies for flow regulation, drought mitigation, and ecological conservation. For instance, rivers like Nir and Viladaragh, with high predictability and constancy, may support more reliable water supply planning, whereas rivers such as Aladizgeh and Dostbeglo, with low predictability, require flexible management approaches to cope with variable flows.

### Limitation and future directions

The study focuses only on rivers in northwestern Iran, and the results may not be directly applicable to other regions with different climatic or topographic conditions. Future research should explore the impact of climate change on river flow seasonality by extending the analysis to longer time periods and examining shifts in Colwell indices. Comparative studies across regions with similar climates and topographies could provide indications into generalized river flow patterns and improve water resource management. Furthermore, analyzing drought and flood spells by incorporating the Colwell indices to evaluate low-flow and high-flow periods, specifically targeting the seasonal variability of river flow in drought-prone regions. Future studies could combine satellite imagery and machine learning to better assess factors affecting river flow changes^57^. In addition, accounting for uncertainty in river flow variability is essential for improving the reliability of these estimates in water resource management^58^.

## Conclusions

In this study, Colwell indices were used as a tool to assess the temporal and spatial changes in river flows. The results related to the Predictability, Constancy, and Contingency indices in the rivers of Ardabil Province reveal significant differences in the flow patterns of this region. According to the results, rivers such as ArbabKandi and Nuran, with the highest Predictability and Constancy values, exhibit more stable flow regimes and are influenced by more consistent rainfall and climatic conditions upstream. Rivers like ArbabKandi and Nuran show high predictability and constancy due to snowmelt-fed flows and minimal human impact, leading to stable and natural flow patterns. Each Colwell index provides unique perspective for water resource management. Predictability indicates the reliability of water availability, helping to guide reservoir operations and water allocation policies. Constancy reflects the stability of flow, which is essential for planning flood control and drought preparedness measures. Contingency captures the seasonality and temporal dependence of flows, informing the design of infrastructure and adaptive management strategies aligned with seasonal water demands and ecological requirements. The results of this study showed the importance of managing surface water resources to maintain the Constancy of river flow regimes. In contrast, rivers like Hir and Deroo, characterized by higher fluctuations in flow rates and lower Constancy and Predictability indices, may be affected by factors such as irregular precipitation, rapid climate changes, or vegetation degradation. Colwell indices (P, C, M) reflect spatiotemporal flow patterns influenced by basin climate and geomorphology. Rivers like ArbabKandi and Nir, with high predictability and constancy, are in basins with moderate slopes, dense vegetation, and stable water sources, resulting in steady flows. In contrast, rivers like Dustbiglu and Atashgah, with low predictability and constancy, lie in steep, sparsely vegetated basins with permeable soils, causing flow variability. These differences showed the role of basin characteristics in flow regime stability and call for further targeted analysis. Trend analysis using the Modified Mann-Kendall test indicates significant decreasing trends in river flow at several stations, particularly Samian, Borran, Dostbeglo, Mashiran, Gilandeh, and Viladaragh (*p* < 0.01). In contrast, stations such as Abgarm, AhleIman, Hir, ShamsAbad, Namin, and Nanakaran show no significant trends (*p* > 0.05). Moderate but significant decreases are detected at stations including ArbabKandi, Barough, Hajahmadkandi, Iril, Nuran, and Nir (*p* < 0.05). These varying trends determined areas vulnerable to climate impacts and can guide strategies to stabilize river flows. This study advances the understanding of river flow seasonality by applying Colwell indices across diverse hydrological regimes in northwestern Iran, offering remarkable indications into spatial and temporal variability shaped by climatic and anthropogenic influences. Our analysis successfully quantified flow predictability, constancy, and contingency, revealing distinct patterns among snowmelt-fed, groundwater-dependent, and rainfall-driven rivers. The methodology provides a transferable framework for assessing river flow seasonality in other semi-arid mountainous regions, offering crucial indication for developing adaptive management strategies to maintain ecological health and water security under increasing climatic and anthropogenic pressures. The results reveal distinct patterns not observed in previous regions. The strong correlation between predictability and constancy (91%) is higher than values reported in temperate European basins (70–80%). The remarkable stability of snowmelt-fed rivers highlights the ability of Colwell’s indices to identify climatic refugia. Additionally, the contrasting predictability trends of minimum and maximum flows provide new hint into how drought and flood vulnerabilities emerge within the same river system. Future work should build on these results to model hydrological responses under projected climate change scenarios. The methodology applied in this study, using Colwell indices for temporal and spatial assessment of river flows, can be effectively transferred and adapted to other semi-arid or topographically diverse regions, aiding broader water resource management under changing climatic conditions. This framework relies on time-series and flow occurrence data and is not directly dependent on climate type, basin scale, or specific geographic conditions. Thus, in theory, it can be applied to other regions with different hydrological settings. However, we acknowledge that confirming this transferability requires empirical testing in basins with diverse characteristics, including arid and semi-arid climates, monsoon systems, glacial basins, and dam-regulated rivers. Comparisons with previous studies in China (Yang et al., 2019), the United States (Poff et al., 2002), and Australia (Thompson et al., 2011) indicate that the behavior of the Predictability, Constancy, and Contingency indices in response to climate change, reduced runoff, dam construction, and human interventions follows a similar pattern. This consistency across regions reinforces the theoretical and empirical validity of the method. Nonetheless, to fully confirm the generalizability and effectiveness of these indices in other hydrological regimes, future studies should apply them in basins with different climatic conditions, compare results with observed or simulated flows in dam-regulated rivers, and evaluate their sensitivity to time-series length, data quality, and land-use change.

## Data Availability

All data generated or analysed during this study are included in this published article.
